# Role of Exercise-Induced Cardiac Remodeling in Ovariectomized Female Rats

**DOI:** 10.1155/2018/6709742

**Published:** 2018-02-13

**Authors:** Renáta Szabó, Zoltán Karácsonyi, Denise Börzsei, Béla Juhász, Amin Al-awar, Szilvia Török, Anikó Magyariné Berkó, István Takács, Krisztina Kupai, Csaba Varga, Anikó Pósa

**Affiliations:** ^1^Department of Physiology, Anatomy and Neuroscience, Faculty of Science and Informatics, University of Szeged, Szeged, Hungary; ^2^Department of Orthopaedics, University of Debrecen, Debrecen, Hungary; ^3^Department of Pharmacology and Pharmacotherapy, University of Debrecen, Debrecen, Hungary

## Abstract

Myocardial extracellular matrix (ECM) is essential for proper cardiac function and structural integrity; thus, the disruption of ECM homeostasis is associated with several pathological processes. Female Wistar rats underwent surgical ovariectomy (OVX) or sham operation (SO) and were then divided into eight subgroups based on the type of diet (standard chow or high-triglyceride diet/HT) and exercise (with or without running). After 12 weeks, cardiac MMP-2 activity, tissue inhibitor of metalloproteinase-2, content of collagen type I, the level of nitrotyrosine (3-NT) and glutathione (GSH), and the ratio of infarct size were determined. Our results show that OVX and HT diet caused an excessive accumulation of collagen; however, this increase was not observed in the trained animals. Twelve weeks of exercise promoted elevation in the levels of 3-NT and GSH and similarly an increase in MMP-2 activity of both SO and OVX animals. The high infarct-size ratio caused by OVX and HT diet was mitigated by physical exercise. Our findings demonstrate that ovarian estrogen loss and HT diet caused collagen accumulation and increased ratio of the infarct size. However, exercise-induced cardiac remodeling serves as a compensatory mechanism by enhancing MMP-2 activity and reducing fibrosis, thus minimizing the ischemia/reperfusion injury.

## 1. Introduction

Premenopausal women have a lower risk of developing cardiovascular disease (CVD) compared to age-matched men; however, this sex advantage for women gradually disappears after the onset of menopause, suggesting that sexual hormones have a strong influence on cardiometabolic parameters [1]. Several physiological changes which develop during menopause may also influence the incidence and manifestation of CVD, such as weight gain, obesity, and its comorbidities [2]. In the pathogenesis of CVD, studies have shown that both estrogen deficiency and obesity contribute to structural and tissue remodeling, as well as to the changes in cardiac function [3–5].

Myocardial extracellular matrix (ECM) serves as an important mediating factor in cardiac development, homeostasis, and remodeling [6]. The most abundant structural components of the ECM are collagens, particularly collagen type I and collagen type III, which are produced primarily by fibroblasts [7], and its synthesis and degradation is essential for normal cardiac structure and function [8]. During pathological conditions, cardiac failure and remodeling are characterized by collagen accumulation, myocyte loss, and impaired rearrangement of cardiac structure [9–11], proving that disruption of ECM/collagen homeostasis is a key factor for the progression of cardiac dysfunction. Degradation of fibrillar collagens and other ECM proteins is catalyzed by matrix metalloproteinases (MMPs), which are a family of zinc-dependent proteases with more than 25 members. MMP-2 is one of the most commonly known among the aforementioned proteins and an enzyme that is constitutively abundant in almost all cell types and characterized by its degrading effect of the denatured collagen (gelatin) and other extracellular matrix proteins [12]. Similar to other enzymes, MMPs are regulated by naturally occurring inhibitors called tissue inhibitors of metalloproteinases (TIMPs), preventing excessive ECM degradation by MMPs [13]. The functional balance between MMPs and TIMPs determines cardiac remodeling [14].

Physical exercise is widely unanimous as a nonpharmacological therapeutic tool for the prevention and treatment of CVD. The latter can initiate cardiovascular adaptations, including reduction in blood pressure, and promotes cardiac remodeling by the development of physiological hypertrophy and reduction of cardiac fibrosis [15, 16]. Furthermore, exercise-mediated cardioprotection has been linked to the activation of antioxidant defense mechanisms and reduction of metabolic risk factors [17]. In our earlier study, we verified that a 12-week voluntary exercise combined with calorie restriction (CR) could attenuate the metabolic parameters, which are frequently linked to major cardiovascular risks in an estrogen-deficient state [18].

We hypothesized that a 12-week voluntary exercise could be an effective strategy in modifying the heart remodeling effect caused in an estrogen-deficient state. Thus, the aim of the current study was to investigate the potential protective effects against the deregulatory and detrimental effects of MMP-2 and collagen content, linked with the detection of necrotic ratio after ischemia/reperfusion injury.

## 2. Materials and Methods

### 2.1. Animals

Female Wistar rats weighing 180–200 g were obtained from Toxi-Coop Zrt., Hungary, and acclimated for at least 1 week prior to experimental use and were maintained under controlled conditions of illumination (12/12 h light/dark cycle) and room temperature (20–23°C). All experimental procedures were performed in accordance with the standards of the European Community guidelines on the care and use of laboratory animals and had been approved by the Institutional Ethics Committee.

### 2.2. Surgery

Following the one-week acclimation period, female Wistar rats of 10 weeks of age were subjected to either ovariectomy surgery (OVX) or sham operation (SO) under anesthesia with thiopental (5 mg/100 g i.p.). During OVX, a bilateral dorsolateral incision was made and the ovaries were removed. On the contrary, the ovaries of SO animals were exteriorized to create similar stress but were not removed. After a 4-week resting period, and to verify the OVX-induced menopause, the serum estrogen levels (Quantikine rat Estrogen ELISA kit, R&D Systems Inc.) were checked using estrogen quantitative enzyme-linked immunosorbent assay (ELISA) [19].

### 2.3. Experimental Design of Dietary Period and Exercise Training

**Figure 1:**
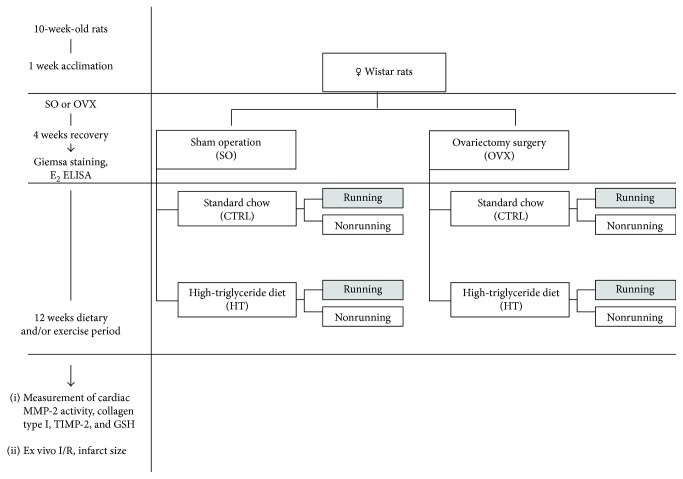
The experimental protocol of the study. SO = sham-operated; OVX = ovariectomized; CTRL = standard chow; HT = high-triglyceride diet.

OVX and SO female rats were randomly divided into 8 new subgroups based on type of diet (standard chow (CTRL) and high-triglyceride diet (HT)) and exercise (with or without running) for 12 weeks. The rats in the CTRL subgroup were fed with a laboratory chow, while the animals in the HT subgroup were subjected to a diet composed of 40% fat content mixed with 60% standard chow. The latter dietary animal groups were further randomly divided into running and sedentary subgroups. The running animals were placed individually into cages fitted with a running wheel, with a free access to the wheel for 24 h per day for 12 weeks. The exercising protocol defined as a voluntary wheel-running model was selected in an effort to isolate the effects of exercising from the additional stress associated with forced exercise protocols [20]. Animals from sedentary subgroups were placed for the same period in standard holding cages. At the end of the experimental period, the estrus phase of SO rats was checked by Giemsa staining to ensure that all SO animals were killed at the same stage of the phase (proestrus phase). All rats were sacrificed, and heart tissues were collected and either mounted into a Langendorff perfusion system to detect ischemia/reperfusion injury ex vivo (10 rats of each group) or were clamped, frozen in liquid nitrogen right after excision, and then stored at −80°C for later use in biochemical analysis (10 rats of each group). The experimental design of the study is shown in Figure 1.

### 2.4. Measurement of MMP-2 Activity

MMP-2 activity was measured from heart samples using gelatin zymography. Fifty micrograms of protein samples were electrophoresed on 8% polyacrylamide gel copolymerized with gelatin (20 mg/ml; type A from porcine skin; Sigma). After electrophoresis, the gels were washed with 2.5% Triton X-100 and incubated for 20 hours at 37°C in an incubation buffer. Staining was performed using 0.05% Coomassie Brilliant Blue followed by destaining with aqueous 4% methanol and 8% acetic acid. A protein ladder (Spectra Multicolor Broad Range Protein Ladder, Thermo Scientific) was used to identify the 2 enzyme isoforms (MMP-2, 72 kDa and 64 kDa). Zymograms were digitally scanned and the intensity of the bands quantified by using Quantity One software (Bio-Rad, Hercules, CA, USA).

### 2.5. Measurement of Total Glutathione (GSH + GSSG)

Heart samples were homogenized in a solution composed of 0.25 M sucrose, 20 mM Tris, and 1 mM dithiothreitol (DTT) and centrifuged at 15,000 ×g for 30 min at 4°C. The supernatant fractions were collected, and then 0.1 M CaCl_2_, 0.25 M sucrose, 20 mM Tris, and 1 mM DTT were added. After incubation at 0°C for 30 min and further centrifugation at 21,450 ×g for 60 min at 4°C, a clear cytosolic fraction was used for enzyme assays. A solution of 125 mM Na phosphate and 6.0 mM EDTA was used as a diluent buffer for the stock solution of glutathione (GSH), glutathione reductase, 5,5′dithio-bis-2-nitrobenzoic acid (DTNB), and *β*-nicotinamide adenine dinucleotide phosphate (*β*-NADPH). A total volume of 40 *μ*l of each blank, standard, or heart sample and equal volumes of DTNB stock solution (20 *μ*l) and *β*-NADPH (140 *μ*l) were added to each well and then incubated at 25°C for 5 min. A 10 *μ*l volume of glutathione reductase was used to start the reaction, and the absorbance was measured at 405 nm in a microplate reader after 10 min from the initiation of the reaction.

In the spectrophotometric assay for total GSH, GSH was sequentially oxidized by DTNB and reduced by NADPH in the presence of glutathione reductase. Total glutathione values were expressed as nmol/mg protein.

### 2.6. Determination of Cardiac 3-NT, Collagen Type I, and TIMP-2

At the end of the 12-week treatment period, the cardiac samples were clamped and frozen after excision. The samples were homogenized (Ultra-Turrax T8; 2 × 30 s) in phosphate buffer (pH 7.4) and then centrifuged at 2000 r.p.m. for 20 min at 4°C. Cardiac 3-NT, collagen type I content, and TIMP-2 were assayed with commercial kits purchased from GenAsia, Shanghai. Optical density was measured at 450 nm (Benchmark Microplate reader; Bio-Rad). Protein content was determined using a commercial protein assay kit (Bio-Rad Labs), and aliquots (20 *μ*l) of the diluted samples (15× or 25× with distilled water) were mixed with 980 *μ*l of distilled water and 200 *μ*l Bradford reagent. After mixing and following 10 min incubation, the samples were assayed spectrophotometrically at 595 nm. Referring to protein values, the cardiac 3-NT levels were defined as pmol/mg protein, collagen type I was defined as pg/mg protein, and TIMP-2 level was expressed as ng/mg protein.

### 2.7. Ischemia/Reperfusion Protocol

After anesthetization, heart tissues were rapidly excised and placed in ice-cold Krebs-Henseleit buffer solution consisting of 11.2 mM glucose, 1.24 mM KH_2_PO_4_, 20.1 mM NaHCO_3_, 119 mM NaCl, 4.7 mM KCl, 1.25 mM CaCl_2_, and 1.24 mM MgSO_4_ and then mounted onto a Langendorff perfusion system. A retrograde perfusion was applied for the hearts via the aorta at constant pressure of 75 mmHg with the Krebs-Henseleit buffer bubbled with 5% CO_2_ and 95% O_2_ at 37°C. After perfusion, local ischemia was induced by occlusion of the left anterior descending coronary artery (LAD) for 30 min, after which it was followed by reperfusion for 120 min. At the end of each experiment, the LAD was reoccluded and perfusion stopped, and the hearts were stained with 1% Evans blue solution injected into the aorta to reveal the area at risk. Heart samples were frozen at −20°C overnight.

### 2.8. Measurement of Infarct Size

Frozen heart-tissue samples were cut into 2 mm thick cross-sectional slices and immersed in 1% 2,3,5-triphenyltetrazolium chloride (TTC) solution prepared in phosphate buffer saline (pH 7.4) for 10 min at 37°C. After TTC staining, the tissue slices were transferred into formalin (10%) solution for 10 min and then placed in phosphate buffer (pH 7.4). Following this incubation, both sides of each slice were photographed with a digital camera. Infarct size was calculated as the percentage of the area at risk.

### 2.9. Statistical Analysis

The results are expressed as means ± S.E.M. Differences between groups were calculated using ANOVA test, and *p* ≤ 0.05 was considered as significant.

## 3. Results

### 3.1. Evaluation of Cardiac MMP-2 Activity in Response to Estrogen Depletion, Exercise, and Nutrition

To better understand the mechanism of how estrogen deficiency, type of diet, and exercise training can influence cardiac fibrosis, we evaluated the activity of 64 and 72 kDa MMP-2 isoforms. At the end of the 12-week experimental period, we found a significantly lower (^∗^*p* < 0.05) activity of 64 kDa MMP-2 in sham-operated (SO/HT) and ovariectomized (OVX/CTRL and OVX/HT) sedentary rats compared with the SO/CTRL group. Exercise training resulted in a significant increase (^#^*p* < 0.05) in the SO/HT and OVX groups compared with nonrunning counterparts. Comparing the MMP-2 activity of running animals, we observed that the high-triglyceride diet in both the SO and OVX animals caused significantly reduced (^∗^*p* < 0.05) values compared with SO/CTRL.

**Figure 2:**
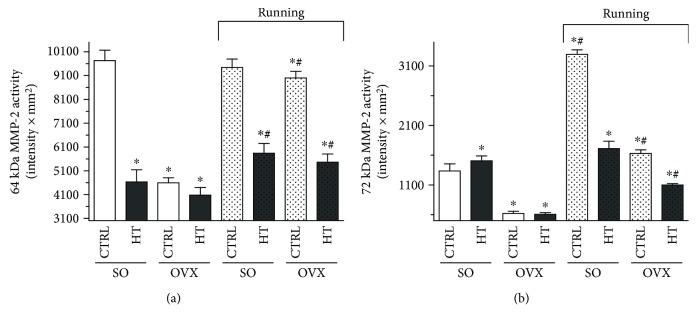
(a) Effects of 12-week wheel-running exercise and nutrition on the cardiac 64 kDa MMP-2 activity (expressed as intensity × mm^2^). Results are shown as means ± S.E.M. *n* = 12. (b) Effects of 12-week wheel-running exercise and nutrition on the cardiac 72 kDa MMP-2 activity (expressed as intensity × mm^2^). Results are shown as means ± S.E.M. *n* = 12. Statistical significance: ^∗^*p* < 0.05 relative to the SO CTRL group, and ^#^*p* < 0.05 shows a significant difference between the running and nonrunning groups. SO = sham-operated; OVX = ovariectomized; CTRL = standard chow; HT = high-triglyceride diet.

The activity of 72 kDa MMP-2 isoform was significantly decreased (^∗^*p* < 0.05) with ovariectomy and high-triglyceride diet (OVX/HT) in sedentary rat hearts. However, voluntary wheel-running exercise caused a significant elevation (^∗^*p* < 0.05) in each SO and OVX subgroup compared with SO/CTRL animals. Hearts from the running SO/CTRL animals exhibited the highest activity of 72 kDa MMP-2 isoform. In addition, significant differences (^#^*p* < 0.05) were observed between running and nonrunning counterparts in the case of the SO/CTRL, OVX/CTRL, and OVX/HT groups. Data are shown in Figures 2(a) and 2(b).

### 3.2. Determination of Cardiac 3-NT Level

**Figure 3:**
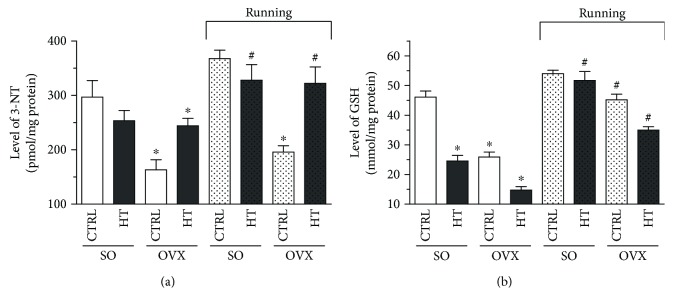
(a) Effects of 12-week wheel-running exercise and nutrition on the cardiac nitrotyrosine (3-NT; expressed as pmol/mg protein). Results are shown as means ± S.E.M. *n* = 6–8. (b) Effects of 12-week wheel-running exercise and nutrition on the glutathione level (GSH; expressed as nmol/mg protein). Results are shown as means ± S.E.M. *n* = 6–8. Statistical significance: ^∗^*p* < 0.05 relative to the SO CTRL group, and ^#^*p* < 0.05 shows a significant difference between the running and nonrunning groups. SO = sham-operated; OVX = ovariectomized; CTRL = standard chow; HT = high-triglyceride diet.

Figure 3(a) shows that cardiac levels of 3-NT were significantly decreased (^∗^*p* < 0.05) in the OVX/CTRL and OVX/HT groups compared with the 3-NT values of the SO/CTRL group. The 3-NT level in the hearts of SO rats decreased with the high-triglyceride diet, but this trend did not reach a statistical significance. Exercise training resulted in a significant increase (^#^*p* < 0.05) in both SO and OVX rats fed with high- triglyceride diet compared with nonrunning counterparts, except in OVX/CTRL rats, in which the 3-NT values were substantially lower (^∗^*p* < 0.05) than in the SO/CTRL group.

### 3.3. Measurement of Cardiac GSH

Cardiac GSH levels were measured at the end of 12 weeks of the experimental period by spectrophotometric assay. A significant decrease (^∗^*p* < 0.05) of GSH was found in heart samples of the sedentary sham-operated (SO/HT) and ovariectomized (OVX/CTRL and OVX/HT) rats compared with the SO/CTRL group. As a result of voluntary exercise training, GSH levels displayed a significant elevation (^#^*p* < 0.05) compared with nonrunning counterparts. Data are presented in Figure 3(b).

### 3.4. Evaluation of Cardiac TIMP-2 Concentration

**Figure 4:**
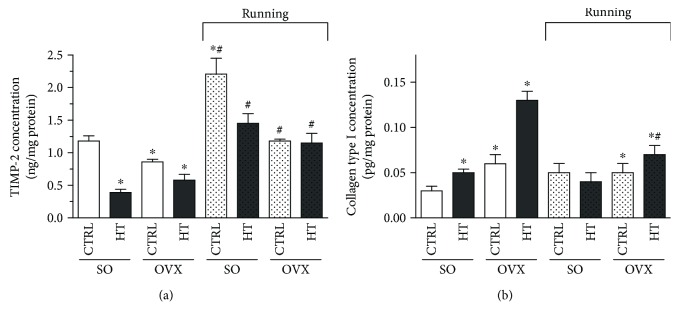
(a) Effects of 12-week wheel-running exercise and nutrition on the cardiac TIMP-2 level (expressed as ng/mg protein). Results are shown as means ± S.E.M. *n* = 5–8. (b) Effects of 12-week wheel-running exercise and nutrition on the cardiac collagen type I accumulation (expressed as pg/mg protein). Results are shown as means ± S.E.M. *n* = 6–9. Statistical significance: ^∗^*p* < 0.05 relative to the SO CTRL group, and ^#^*p* < 0.05 shows a significant difference between the running and nonrunning groups. SO = sham-operated; OVX = ovariectomized; CTRL = standard chow; HT = high-triglyceride diet.

To determine the role of the MMP/TIMP system on ECM turnover, cardiac TIMP-2 was also examined. Our results reveal that exercise training significantly enhanced the TIMP-2 values, which was diminished by estrogen depletion and high-triglyceride diet. Significant elevation (^#^*p* < 0.05) was noted between the running and nonrunning counterparts. Data are shown in Figure 4(a).

### 3.5. Concentration of Cardiac Collagen Type I

To test the hypothesis that exercise training might modulate the accumulation of fibrotic tissue, cardiac collagen type I was measured by ELISA. While estrogen withdrawal and high-triglyceride diet resulted in an excessive accumulation of collagen type I, physical exercise training significantly (^#^*p* < 0.05) reduced the collagen accumulation in the heart of OVX rats fed with high-triglyceride diet. Data are presented in Figure 4(b).

### 3.6. Myocardial Infarction Extension

**Figure 5:**
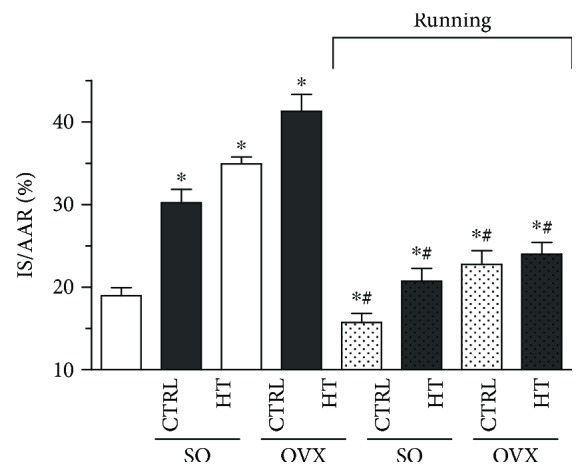
Effects of 12-week wheel-running exercise and nutrition on the extension of myocardial infarction. Infarct size is demonstrated as a percentage of the area at risk. Results are shown as means ± S.E.M. *n* = 8–10. Statistical significance: ^∗^*p* < 0.05 relative to the SO CTRL group, and ^#^*p* < 0.05 shows a significant difference between the running and nonrunning groups. SO = sham-operated; OVX = ovariectomized; CTRL = standard chow; HT = high-triglyceride diet.

Figure 5 clearly shows that estrogen depletion and high-fat diet significantly increased (^∗^*p* < 0.05) the necrotic extension of the heart myocardial infarction compared with the SO/CTRL group. However, 12 weeks of exercise abolished the detrimental effects of ovariectomy and high-triglyceride diet with a significant reduction of infarcted area in each running group.

## 4. Discussion

Cardiovascular disease (CVD) is one of the major causes of death, and it can lead to heart failure including cardiac remodeling, cardiac apoptosis, and fibrosis. Inflammation, disruption of antioxidant states, and estrogen depletion are the risk factors that can lead to cardiac hypertrophy. Exercise training which is a safe nonpharmacological therapeutic tool for the prevention and treatment of CVD promotes beneficial effects, such as decreased aging-induced cardiomyocyte apoptosis, decreased risk of heart failure, and improved cardiac function. A growing number of studies have addressed that alterations in the tightly regulated ECM homeostasis can have a profound influence on the structure and function of the heart [8, 21]. Cardiac fibrosis is characterized by an excessive deposition of ECM proteins, especially collagens, leading to a pathological remodeling with increased myocardial stiffness, hypertrophy, and acute cardiac injury such as myocardial infarction [10]. Increased deposition of interstitial collagen is resulted from aging [22], myocardial ischemia [23], inflammatory processes [24], diabetes [25, 26], and hormones [27]. It has emerged that sexual hormones and their receptors play a key role in the regulation of ECM proteins. The interplay of MMP-2, peroxynitrite (ONOO^−^), and glutathione (GSH) in heart tissue shows new insight into the pathophysiology of the heart in estrogen-deficient conditions.

We examined the mechanism of estrogen depletion-induced collagen accumulation and fibrosis, which may occur through MMP-2 downregulation and cardiac hypertrophy. Our results clearly show that estrogen withdrawal and high-triglyceride diet caused a significant increase of collagen content and reduced cardiac levels of 3-NT and GSH. However, 12 weeks of moderate physical exercise could attenuate the OVX-induced heart fibrosis via GSH/3-NT and MMP-2 regulation. Our previous findings proved that elevation of blood pressure caused by OVX may participate in these changes [1, 28]. The antioxidant effects of endogenous and exogenous estrogen may play a critical role in eliciting vasoprotective effects, the oxidative stress increased in postmenopausal women and in animals [29, 30]. Pedram et al. showed that estrogen prevents against myofibroblast development and production of collagen and fibronectin proteins that cause cardiac remodeling [4], while E_2_ reduces the turnover of ECM and exerts protective effects against cardiomyocyte apoptosis, estrogen withdrawal leading to left ventricular hypertrophy, collagen deposition, and increased sensitivity to constrictive agents, such as angiotensin II [31]. In accordance with the literature, our findings show that ovariectomy caused damage to cardiac morphology with collagen type I content enhancement. Fibrosis is multifactorial, and the molecular mechanisms related to the regulation of ECM metabolism involve multiple signaling pathways. It is widely accepted that inactivity or sedentary lifestyle, reduction in circulating estrogen level, aging process, and oxidative stress can cause excessive accumulation of collagen matrix and increased progression of cardiac dysfunction [32]; however, the mechanism responsible for the reduction of cardiac fibrosis induced by physical training is not fully identified. In this current study, we focused on the role of voluntary physical exercise and the type of diet in the modifying effects of MMP-2 regulation. MMPs and their tissue inhibitors regulate the profile of ECM both in normal and pathological conditions, so the balance between MMPs and TIMPs determines cardiac remodeling [33]. In a previous study, Felix et al. proved that ovarian hormone deprivation caused significant damage to cardiac morphology, whereas the low intensity of aerobic exercise prevented the increase in cardiac fibrosis. However, they did not examine the regulatory effects of MMP-2 [34]. Kwak et al. investigated the alterations of collagen profile in response to exercise training and proved that physical training protected against age-related downregulation of active MMPs [15]. Our research group recently demonstrated that a 12-week voluntary exercise significantly increased the MMP-2 activity, indicating the protective effects of exercise against collagen accumulation and fibrosis. MMP-2 degrades the ECM proteins which are responsible for cardiac remodeling. In addition, we also found that exercise training caused a reduction in collagen type I content and improved the MMP/TIMP profile resulting in protective effects against cardiac injuries. In the present study, 12 weeks of exercise training significantly decreased the infarct size, mitigating the estrogen withdrawal, high-triglyceride diet, and obesity-related enhanced ratio of necrotic area after ischemia/reperfusion. Many studies support the notion that women in menopausal and postmenopausal periods have greater risk for CVDs, including myocardial infarction (MI), which is related to an increase in oxidative stress and a reduction in nitric oxide (NO) bioavailability [35, 36]. Physical exercise has become a nonpharmacological therapeutic option in the prevention and treatment of CVD in both women and men. Exercise-induced improvement in myocardial capillarization, intracellular redox balance, and endothelial dysfunction by increasing of NO production can minimize the ratio of infarct size [37, 38]. Almeida et al. reported that exercise training decreased the protein expression of one of the main pathways generating ROS and also increased the antioxidant enzyme catalase, which contributed to improvement in cardiac function and remodeling process in ovariectomized rats after MI [31]. Clinical findings also demonstrated that cardiac fibrosis is strongly associated with obesity and contributes to cardiac dysfunction in obese women. Kosmala et al. concluded that abnormalities of the left ventricular function are related to the changes in the MMP/TIMP system that might promote the attenuation of ECM degradation, mainly due to the downregulation of MMP-2 in obese women [3]. Our experimental protocol with exercise training in both sham-operated and ovariectomized rats resulted in a significant increase in the MMP-2 activity and diminished the collagen accumulation in the heart, representing an important protective strategy to treat cardiac pathologies. The mechanism related to exercise-induced collagen turnover and cardioprotection is due to the MMP/TIMP profile and the result of the activation pathways of MMP. MMP-2 can be activated by proteolytic and nonproteolytic ways. Proteolytic activation of the 72 kDa zymogen occurs by removal of its autoinhibitory propeptide to render an active 64 kDa MMP-2 or by posttranslational modification caused by peroxynitrite (ONOO^−^) in the presence of cellular glutathione (GSH) [39]. The ability of ONOO^−^, which is a product of superoxide anion and nitric oxide (NO), to suppress [40, 41] or activate [39] MMP-2 is controversial. Rajagopalan et al. found that ONOO^−^ enhanced the gelatinolytic activity of unpurified MMP-2 in smooth muscle cells [42]. Oxidative stress-induced posttranslational modifications can result in the activation of MMP-2. The presence of the intracellular level of glutathione and ONOO^−^ causes S-glutathiolation and conformational changes and results in an active MMP form [43].

GSH is a nonenzymatic antioxidant in the cells, and its depletion is considered as an important biomarker of oxidative stress [44]. A significant reduction of the GSH level was noted in our present study in response to estrogen withdrawal and high-triglyceride diet. The duration of 12 weeks of exercise training could restore the GSH level of the heart in agreement with others [44, 45]. GSH plays a critical role in cardiac function, maintaining redox homeostasis [17]. Frasier et al. demonstrated that physical exercise preserves cardiac glutathione pools and decreases myocardial damage after ischemic insult [46].

The complexity of functional properties of MMPs poses some limitations, and the activation of MMP-2 is not uniformly concordant, especially in cardiovascular pathologies. Moreover, there are no similar studies which investigated the role of lifestyle changes (estrogen withdrawal, exercise, and type of diet) in the activation and regulation of MMP-2.

In conclusion, the 12 weeks of exercise caused enhancement in the levels of cardiac 3-NT and GSH. Elevation in 3-NT and GSH levels with activation of 72 kDa MMP-2 may play a compensatory role against cardiac fibrosis. These data are in line with our further findings that exercise-induced activation of MMP-2 and the improved balance between MMP and TIMP contribute to cardioprotection and serves as a therapeutic agent in cardiac remodeling.
